# Sterols and Triterpenes: Antiviral     Potential Supported by In-Silico Analysis

**DOI:** 10.3390/plants10010041

**Published:** 2020-12-26

**Authors:** Nourhan Hisham Shady, Khayrya A. Youssif, Ahmed M. Sayed, Lassaad Belbahri, Tomasz Oszako, Hossam M. Hassan, Usama Ramadan Abdelmohsen

**Affiliations:** 1 Department of Pharmacognosy, Faculty of Pharmacy, Deraya University, Universities Zone, P.O. Box 61111, New Minia City, Minia 61519, Egypt; noura_shady2013@yahoo.com; 2 Department of Pharmacognosy, Faculty of Pharmacy, Modern University for Technology and Information, Cairo 11865, Egypt; khayrya.youssif@gmail.com; 3 Department of Pharmacognosy, Faculty of Pharmacy, Nahda University, Beni-Suef 62513, Egypt; ahmedpharma8530@gmail.com (A.M.S.); abuh20050@yahoo.com (H.M.H.); 4 Laboratory of Soil Biology, University of Neuchatel, 2000 Neuchatel, Switzerland; lassaad.belbahri@unine.ch; 5 Departement of Forest Protection, Forest Research Institute, 05-090 Sękocin Stary, Poland; T.Oszako@ibles.waw.pl; 6 Department of Pharmacognosy, Faculty of Pharmacy, Beni-Suef University, Beni-Suef 62514, Egypt; 7 Department of Pharmacognosy, Faculty of Pharmacy, Minia University, Minia 61519, Egypt

**Keywords:** sterols, triterpenes, antiviral potential, SARS-CoV-2

## Abstract

The acute respiratory syndrome caused by the novel coronavirus (SARS-CoV-2) caused severe panic all over the world. The coronavirus (COVID-19) outbreak has already brought massive human suffering and major economic disruption and unfortunately, there is no specific treatment for COVID-19 so far. Herbal medicines and purified natural products can provide a rich resource for novel antiviral drugs. Therefore, in this review, we focused on the sterols and triterpenes as potential candidates derived from natural sources with well-reported in vitro efficacy against numerous types of viruses. Moreover, we compiled from these reviewed compounds a library of 162 sterols and triterpenes that was subjected to a computer-aided virtual screening against the active sites of the recently reported SARS-CoV-2 protein targets. Interestingly, the results suggested some compounds as potential drug candidates for the development of anti-SARS-CoV-2 therapeutics.

## 1. Introduction

**Figure 1:**
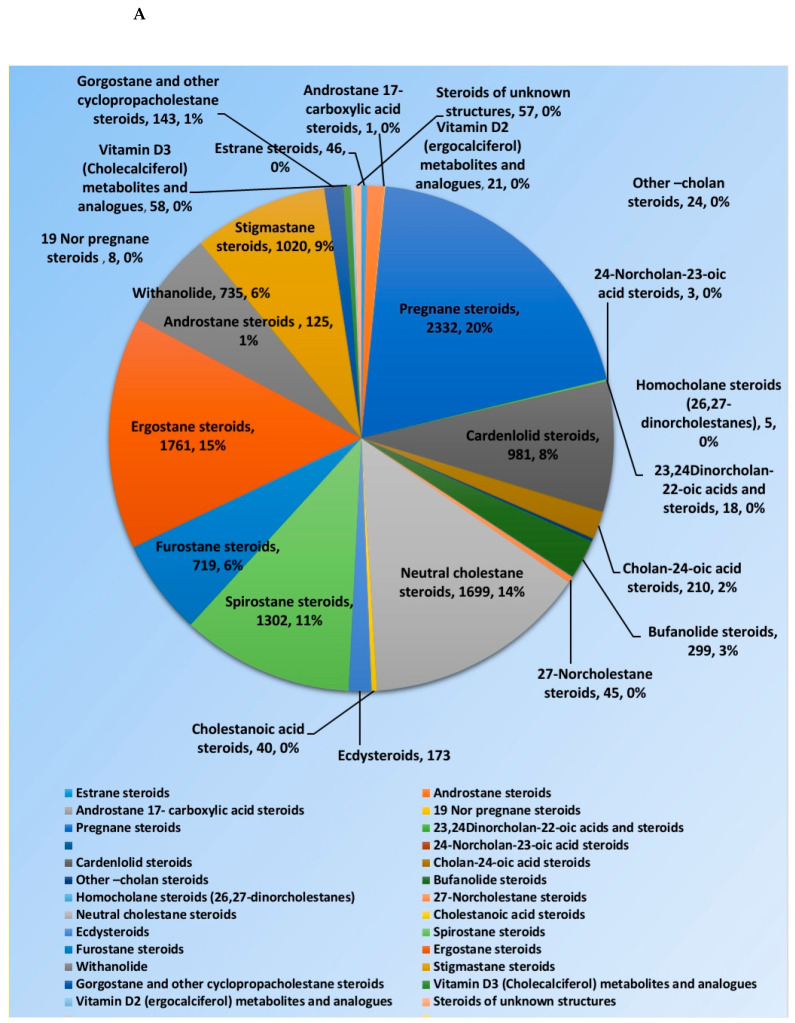

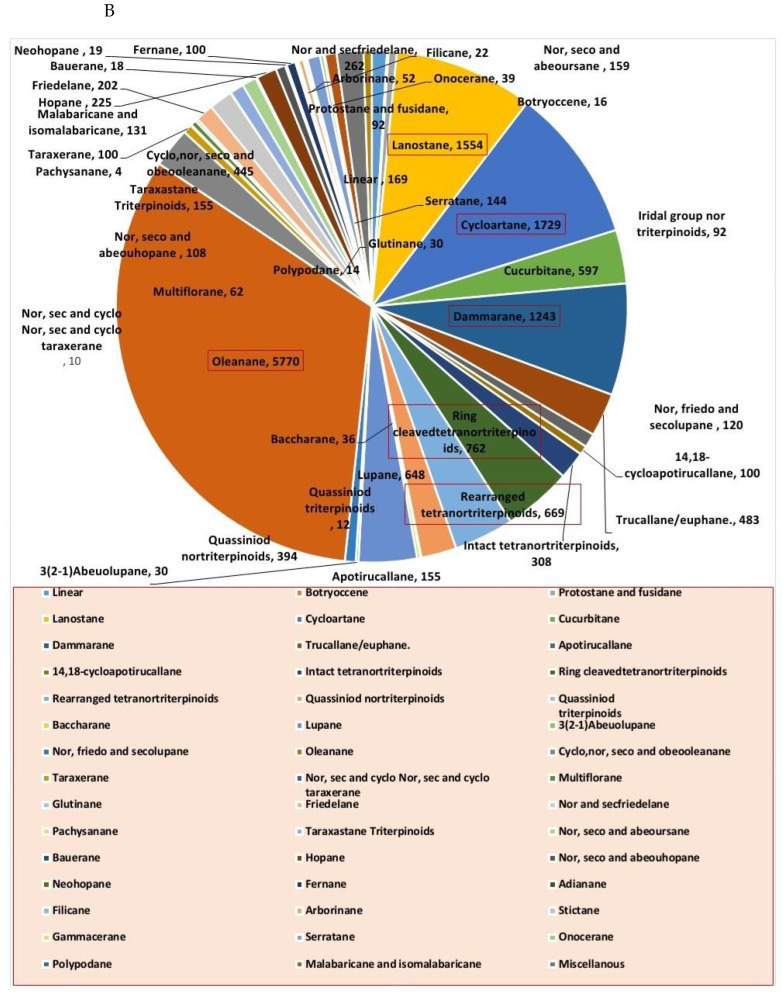
Pie charts indicating the distribution of chemical subclasses of steroids (**A**) and triterpenes (**B**).

Steroids and triterpenes are considered common natural product classes that are widespread in different marine and terrestrial natural sources (e.g., plants, animals, and microorganisms) [1,2,3]. Additionally, they comprise many sub-classes with enormous chemical diversity. These two classes of compounds have been provided several successful drugs for various ailments since the discovery of digoxin in 1785, like cortisol, fusidic acid, carbenoxolone, and β-Aescin [4]. These compounds possess a myriad of biological activities, digoxin used in the treatment of heart failure and atrial fibrillation [5], fusidic acid used as topical antibiotics [6]. Carbenoxolone enhances peripheral insulin sensitivity [7] and treatment of gastric ulcer [8] and β-Aescin used in the treatment of human hepatocellular carcinoma SMMC-7721 cells [9]. Steroids and triterpenes possess various potential antiviral properties such as anti-Herpes simplex virus activity [10], anti-hepatitis B activity [11], anti-HIV1 and 2, AIDS, and hepatitis C virus activities [12]. Steroids class consists of 25 chemical subclasses with about 11,825 compounds that have been previously reported [13] (Figure 1A) While, triterpenoids class of compounds consists of 47 chemical subclasses with about 18,864 chemical compounds that were previously, isolated, and identified from different natural sources as shown in (Figure 1B).

**Figure 2:**
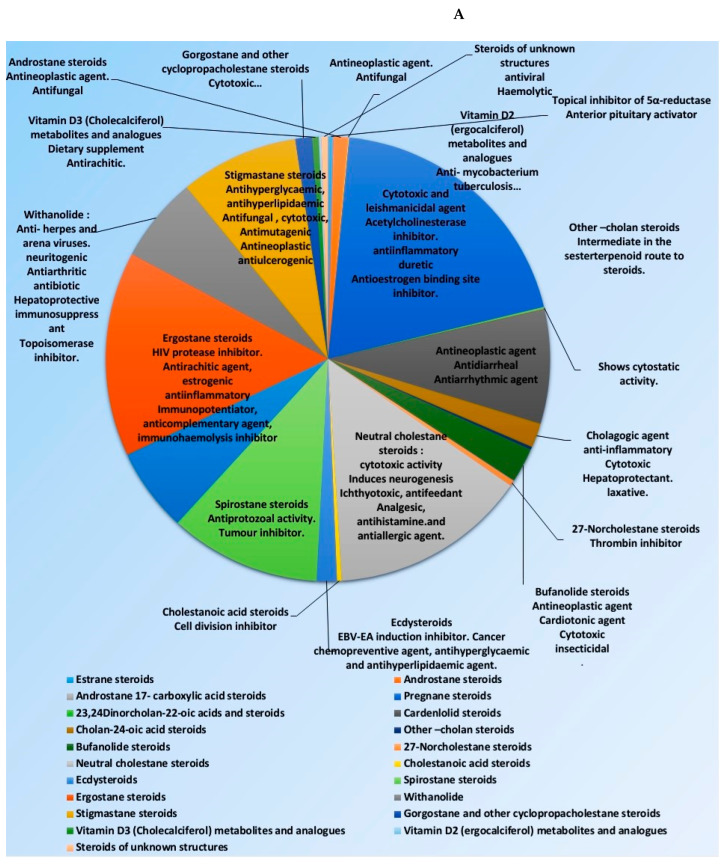

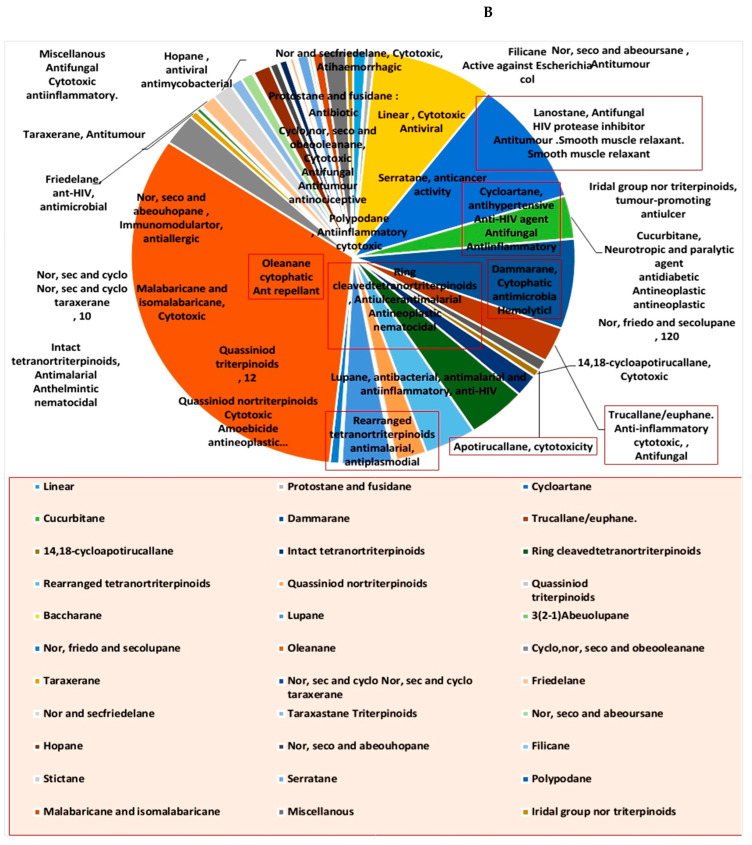
Pie charts indicating the biological activities of steroids (**A**) and triterpenes (**B**).

Many representatives from both classes (i.e., steroids and triterpenoids) have been reported for a wide spectrum of pharmacological activity (e.g., antimicrobial, anticancer, and anti-inflammatory activities) (Figure 2). Our current review aims to shed light on the antiviral potential of these interesting secondary metabolites classes (i.e., steroids and triterpenes) illustrating their mode of actions either directly (i.e., direct viral inhibition) or indirectly (i.e., boosting the host immune response, reducing the inflammatory and antagonize some host-based molecular targets used by viruses for cell entry and multiplication). We also investigated the probability of these reviewed triterpenes and sterols to provide promising candidates for the development of effective therapeutics towards the current SARS-CoV-2 pandemic by molecular docking experiments against the currently available protein targets (either viral or human-based targets).

## 2. Methodology

### 2.1. Databases

The review article search was conducted in the following databases: Research gate (https://www.researchgate.net/search), Science Direct (https://www.sciencedirect.com), PubMed (https://pubmed.ncbi.nlm.nih.gov), Scopus (https://www.scopus.com), Web of Science (https://clarivate.libguides.com/webofscienceplatform/alldb), Google Scholar (https://scholar.google.com), and DNP (Dictionary of Natural Products) database. The keywords “antiviral steroids and triterpenes” were paired with “natural products”, “marine drugs”, “medicinal plants”, “herbal drugs”, “crude extracts”, “fungi”, or “synthetic derivatives of natural products” to obtain published records till 2020. 

### 2.2. Molecular Docking

Docking study was carried out against all available crystal structure (Figure 2) of SARS CoV-2 proteins (3 proteins) in Protein Data Bank (PDB) (along with other four human proteins (4 proteins) involved in the entry and possessing of the virus (Table 1) using Autodock Vina docking machine [14]. The viral crystal structures are SARS-CoV-2 main protease (M^pro^) (PDB ID: 6LU7), papain-like protease (PL^pro^) (PDB ID: 6WXR)) which are key viral cysteine proteases essential for the viral replication inside the host cell, and viral ADP ribose phosphatase (ARP) (PDB ID: 6W02) which is essential for the viral protection against the ADP-ribosylation activated by the innate immune system of the host cells. The four human targets are adaptor protein 2 associated kinase.

(AAK1) (PDB ID: 4WSQ) and cyclin G-associated kinase (GAK) (PDB ID: 4Y8D) are two important proteins for the viral entry into the host cell [15]. Moreover, the two host-based proteases furin (PDB ID: 6EQX) and cathepsin L (PDB ID: 2YJC) that are involved in viral spike protein (S-protein) activation).

The applied docking protocol deals with the protein as a rigid structure and the tested compound as a flexible structure during its computations. The co-crystallized ligands were used to determine the binding sites. The ligand-to-binding-site shape matching root means square (RMSD) threshold was set to 2.0 Å. The interaction energies were determined using the Charmm Force Field (v.1.02) with 10.0 Å as a non-bonded cutoff distance and distance-dependent dielectric. Then, 5.0 Å was set as an energy grid extending from the binding site. The tested compounds were energy-minimized inside the selected binding pocket. The editing and visualization of the generated binding poses were performed using Pymol software [16].

## 3. Findings and Discussion

### 3.1. Anti-HIV Agents

Sterol and triterpenes reported as anti-HIV candidates have been reported extensively in the literature, where they exhibited multiple modes of action as antiviral.

#### 3.1.1. Reverse Transcriptase Inhibitors

Reverse transcriptase is a key enzyme in retroviruses like HIV (i.e., essential for the viral replication process), and hence it has been extensively studied for the development of anti-HIV therapeutics. Several sterols and triterpenes have been reported as potent HIV reverse transcriptase inhibitors during the last 20 years.

Stigmastanol (**1**) is a common sterol in many plants and present in large quantities in rice bran, where it can be easily isolated and purified. This sulfated sterol has been reported to inhibit HIV-’s reverse transcriptase competitively with IC_50_ = 3.9 μM. [17]. Clathsterol (**2**) the major sterol in *Clathria* species has been found to inhibit HIV-1 reverse transcriptase activity at a concentration of 10µM [18]. Furthermore, concise synthesis of the clathsterol steroidal core has also been explained [19].

**Figure 3:**
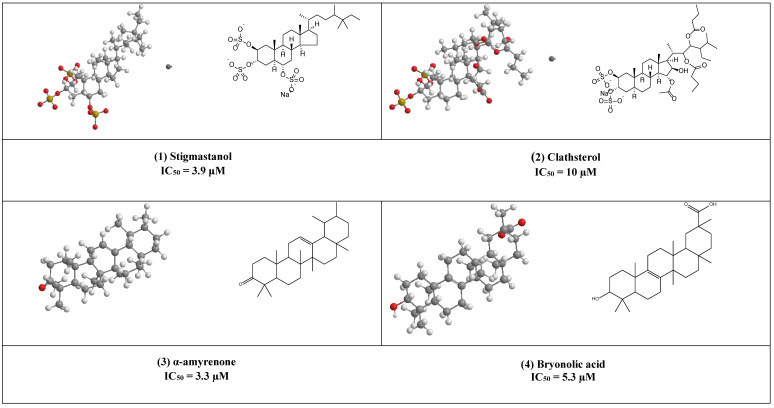

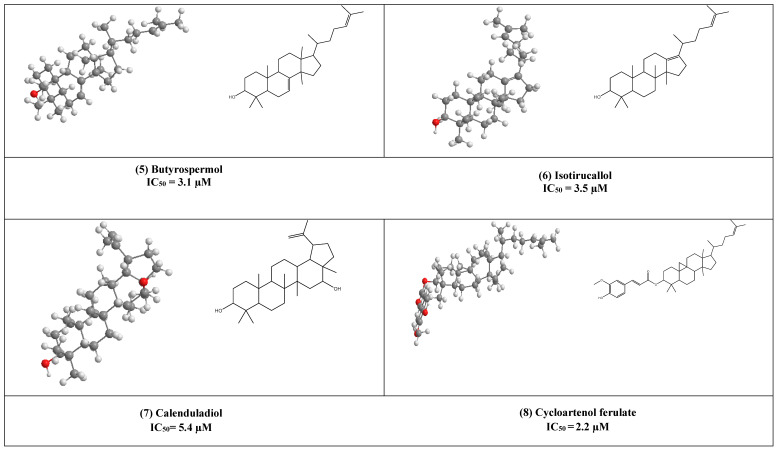

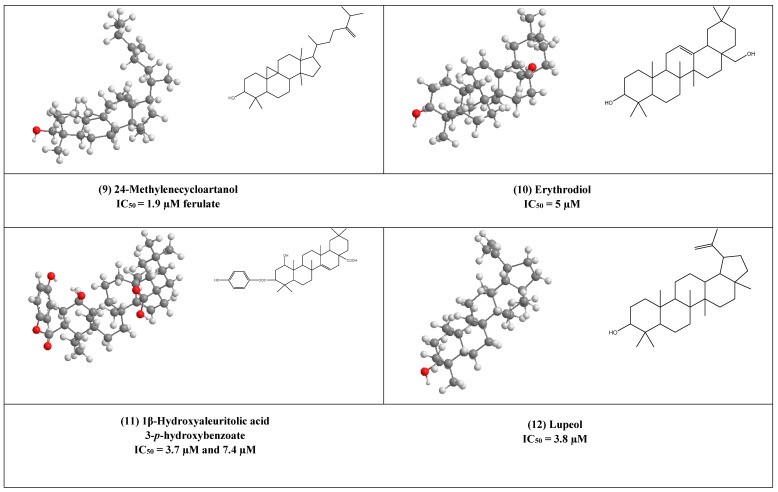

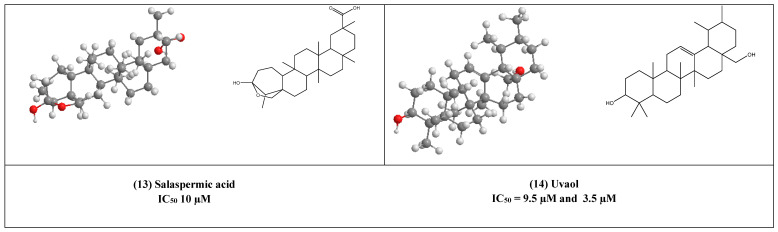
The 2D and 3D chemical structures of virus reverse transcriptase inhibitors.

Both α, β- amyrenone (3) are triterpene derivatives of the ursane and oleanane series, as shown in (Figure 3). They were commonly isolated from Burseraceae family species such as *Protium heptaphyllum*, *Protium. opacum* var. *opacum*, *Protium. giganteum*; *Trattinnickia glaziovii*, and *Trattinnickia. peruviana*. 

Both compounds have been reported to possess several pharmacological effects (e.g., anti-inflammatory, antinociceptive activities together with lipase, α-glucosidase, and α-amylase, inhibitory activities) [20]. In addition, α-amyrenone has also shown strong virucidal activity against HIV-1 reverse transcriptase in a low IC_50_ value equal to 3.3 µM.

Bryonolic acid (4) is a pentacyclic triterpenoid found in the family Cucurbitaceae. It has many remarkable potentials for the treatment of different diseases (e.g., allergic and inflammatory diseases) [21,22]. Additionally, it has shown anti-HIV activity by inhibiting the reverse transcriptase enzyme (IC_50_ 5.3 µM) [23].

Butyrospermol (5) and Isotirucallol (6) are euphane and dammarane types triterpenes, respectively (Figure 3). Both compounds have been reported to be major metabolites in *Camellia japonica* and *Camellia. sasanqua*. Moreover, they were able to inhibit HIV-1 reverse transcriptase with IC_50_ values of 3.1 µM and 3.5 µM, respectively [17].

Another potent inhibitor of the HIV-1 reverse transcriptase enzyme is calenduladiol (7), which is a lupine type pentacyclic triterpene that found in *Calendula officinalis.* Calenduladiol could potentially inhibit the viral HIV-1 reverse transcriptase with IC_50_ equal to 5.4 µM.

Two active cycloartane type pentacyclic triterpenes compounds of rice bran, cycloartenol ferulate (8), and 24-Methylenecycloartanol ferulate (9) displayed potent anti-HIV through competitive inhibition of reverse transcriptase with (IC_50_ 2.2 µM and 1.9 µM, respectively) [17].

Erythrodiol (10), is the precursor of pentacyclic triterpenic acids that were isolated from *Olea europaea*. This compound has displayed antiviral activity against HIV-1 reverse transcriptase with an IC_50_ value of 5 µM. 

1β-Hydroxyaleuritolic acid 3-*p*-hydroxybenzoate (11), a pentacyclic triterpene as shown in (Figure 1), was isolated from *Maprounnea Africana*. The compound is considered a potent inhibitor of HIV-1 reverse transcriptase with IC_50_ 3.7 µM [24].

Lupeol (12) is a pentacyclic triterpene that is widely distributed in edible vegetables and fruits. Research over about three decades has displayed numerous remarkable pharmacological activities of lupeol. Several in vitro and preclinical animal studies have suggested potential anti-inflammatory, immunomodulatory, antiproliferative, and cholesterol-lowering agents [25,26,27,28,29,30,31]. The anti-inflammatory and immunomodulatory activity of this compound has been linked to its regulation of several cytokines expression (e.g., like IL-2, IL4, IL5, Ilβ), [30], and hence it can help as an adjuvant therapeutic agent in the treatment of COVID-19 to control the cytokine storm associated with this pandemic disease [32].

Furthermore, Akihisa et al. reported that lupeol strongly can inhibit HIV-1 reverse transcriptase in IC_50_ value of 3.8 µM [16].

Salaspermic acid (13), and oleanane type triterpene derivative that has been found to act as an inhibitor of HIV reverse transcriptase in H9 lymphocyte cells (IC_50_ 10 µM). This triterpene has been reported as the major metabolite of the roots of *Triterygium wilfordii* [33,34].

Uvaol (14), an olive-derived lupine type pentacyclic triterpene, was also found to inhibit HIV maturation through inhibiting HIV-1 reverse transcriptase in IC_50_ value of 9.5 µM [35].

#### 3.1.2. Protease Inhibitors

Proteases are common viral hydrolytic enzymes. They are usually involved in viral entry and initiation of the viral replication process. Additionally, other human-based proteases are utilized by the majority of infectious viruses in their host cell attachment and entry [36]. Thus, pan-protease inhibitors (e.g., camostat) have shown broad-spectrum antiviral activity including the currently spread SARS-CoV-2 [36].

**Figure 4:**
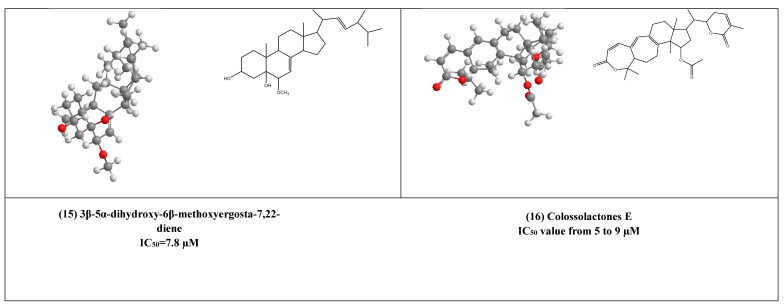

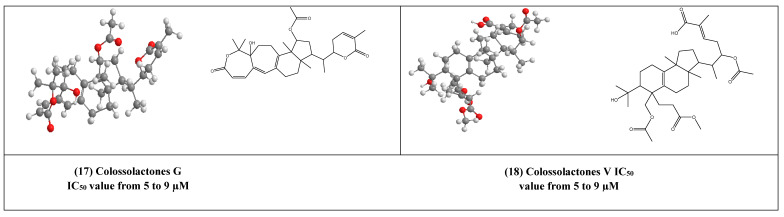
The 2D and 3D chemical structures of virus protease inhibitors.

Some lanostane-type triterpenes isolated from the medicinal mushrooms *Ganoderma* species have been shown in vitro inhibitory effects against HIV-1 protease. For example, 3β-5α-dihydroxy-6β-methoxyergosta-7,22-diene (15) (Figure 4) [37] was isolated from an edible mushroom species called *G. lucidum* and was reported as a potent non-competitive HIV-1 protease inhibitor (IC_50_ = 7.8 µM). Additionally, three *G.colosum*-derived lanostane type triterpenes namely, colossolactones E, G, and V (16–18) (Figure 4) [38] as having been displayed anti-HIV-1 protease with IC_50_ values ranged from 5 to 9 µM, respectively.

In addition to being a potent HIV reverse transcriptase [34], uvaol (14) was also found to inhibit HIV-1 protease with an IC_50_ value of 3.5 µM [35].

#### 3.1.3. DNA Polymerase Inhibitors

The *Maprounnea africana*-derived pentacyclic triterpene,1β-hydroxyaleuritolic acid 3-*p*-hydroxybenzoate (11) has been also revealed potent inhibitory activity toward DNA polymerase with IC_50_ equal to 7.4 µM in addition to its anti-HIV-1 reverse transcriptase activity [24].

### 3.2. Replication Inhibitors:

β-Aescin (19) is a pentacyclic triterpene that was isolated from *Aesculus hippocastranum* (Hippocastanaceae) seed extract. β-Aescin was reported to exhibit anti-oedematous, anti-inflammatory, and antioxidative effects [39,40]. It has been reported that β-Aescin inhibits NF- *ƙ*B activation and cytokines production in LPS-treated mice and numerous tumor cells, macrophages, and endothelial cells [41,42]. Additionally, this inhibition was also found to play an important role in inhibiting Herpes Simplex virus-1 (HSV-1) replication with an IC_50_ value of 1.5 and 2.4 µM was calculated in Human corneal-limbal epithelial (HCLE) and normal human conjunctival epithelial cell line (NHC) cells. The virucidal activity of β-aescin could be due to its ability to interfere with cell membranes and cholesterol homeostasis. 

Moreover, this glycosylated triterpene (i.e., β-aescin) has been also reported to inhibit the replication of SARS-CoV-1 in vitro (IC_50_ 6 µM).

Asiatic acid (20) is a naturally occurring aglycone of ursane-type pentacyclic triterpenoids. It has been previously reported from a wide range of plants e.g., *Actinida argute* roots, *Centella Asiatica* leaves, and *Combretum laxum* Stems [43]. It has poly-pharmacological properties like antioxidant, anti-inflammatory. Moreover, it affects and regulates apoptosis that contributes to its therapeutic effects in various diseases. Numerous in vivo and in vitro studies reported that asiatic acid (20) influences many enzymes, receptors, growth, and transcription factors, in addition to several cell signaling cascades. Moreover, it inhibits both HIV-1 and enterovirus 71 (EV71) replications in acutely infected host cells with IC_50_ equal to 9 and 20 µM, respectively [43,44].

The triterpene lactone lancilactones C (21), which was isolated from the roots of *Kadsura lancilimba* (Schizandraceae), has been inhibited HIV replication with an IC_50_ value of 1.4 µM [45]. 

Oleanolic acid (OA) (22) is a pentacyclic triterpenoid that possesses a variety of interesting biological activities (e.g., anticancer, antimicrobial, and anti-inflammatory activities) [46]. This common triterpene has been previously reported from more than 1600 different plant species [47,48]. OA has been found to inhibit HIV-1 replication in acutely infected H9 lymphocyte cells (IC_50_ 7.5µM) [44,49]. synthesized derivatives of OA by the modification of C12-C13 double bond yielding a series of new compounds that was 3-fold more active than OA [49].

**Figure 5:**
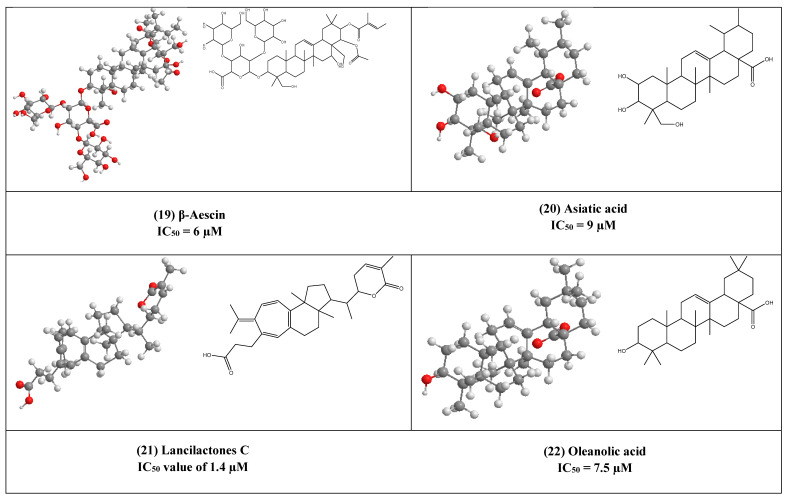

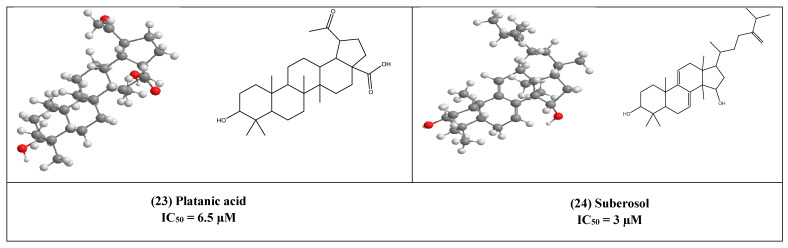
The 2D and 3D chemical structures of virus replication inhibitors.

Platanic acid (23) is a pentacyclic triterpenoid as shown in (Figure 5), that was isolated from the leaves of *Syzigium claviflorum* (Myrtaceae) [33]. It showed an inhibitory effect on HIV replication with an IC_50_ of 6.5 µM, while its IC_50_ for inhibition of uninfected H9 cell growth was 90 µM. 

Salaspermic acid (13) is an oleanane-type triterpene derivative, which was isolated from the roots of *Triterygium wilfordii* [33,34]. It was previously mentioned to have inhibitory activity against HIV reverse transcriptase. Moreover, it has an anti-HIV replication in H9 lymphocyte cells with an IC_50_ value of 10 µM. 

Another lanostane-type triterpene namely, suberosol (24) has shown inhibitory activity against HIV replication in H9 lymphocyte cells with IC_50_ equal to 3 µM. This compound has been previously isolated from *Polyalthia suberosa* (Annonaceae) [50].

## 4. Other Compounds with Antiviral Activity

**Figure 6:**
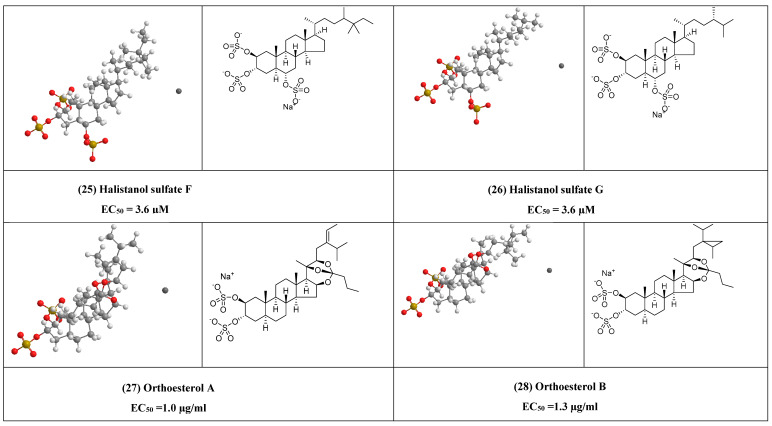

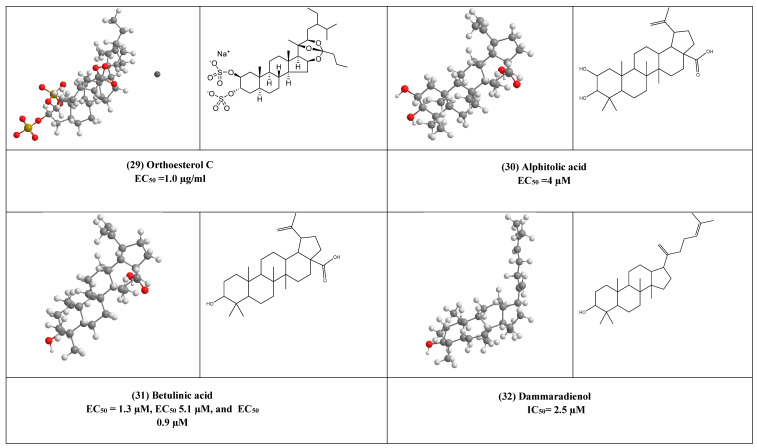

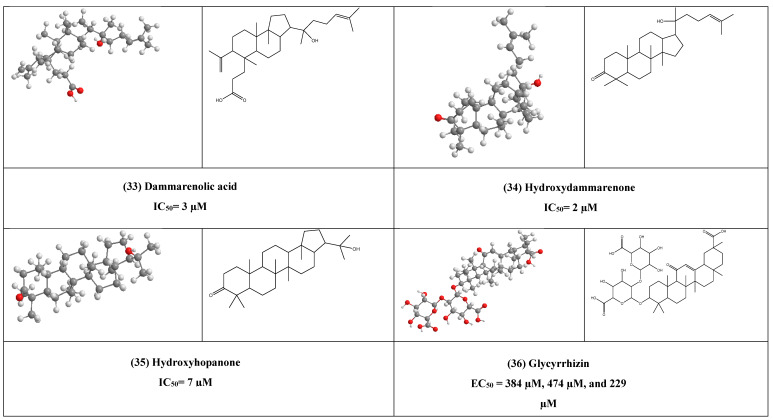

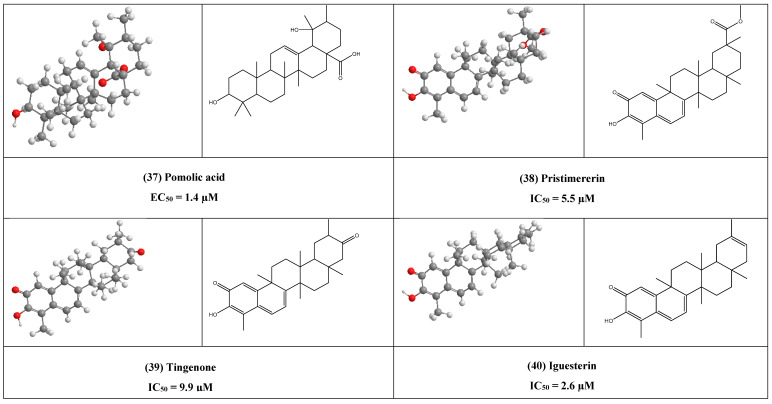

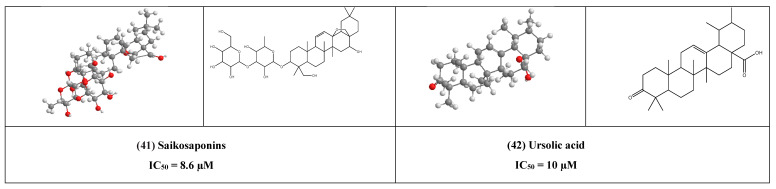
The 2D and 3D chemical structures of other antiviral compounds.

Halistanol sulfate F (25) and halistanol sulfate G (26) (Figure 6) are two steroid sulfate oxoanions that exhibited antiviral activity against HIV-1, with EC_50_ = 3.6 µM, respectively, and were previously isolated from *Psedaxinissa digitate* [51]. 

Additionally, orthoesterol A, B and C disulfate (27–29) (Figure 6) were sterol disulfate orthoester, were previously, isolated from the marine sponge *Petrosia weinbergi* and showed activities against feline leukemia virus in vitro (EC_50_ = 1.0, 1.3, and 1.0 µg/mL), respectively [52]. 

Alphitolic acid (30) (Figure 6) is an ursan-type pentacyclic triterpene, that was isolated from *Rosa woodsii* (F. Rosaceae). It has shown anti-HIV activity with EC_50_ of 4 µM [44]. Moreover, it exhibits a strong inhibition against acute inflammation induced by carrageenan and xylene, this effect is quite similar to that of dexamethasone. Studies suggested that alphitolic acid anti-inflammatory activity may be due to inhibition of the nuclear factor kappa-B (NF-κB), or suppressing the secretion of pro-inflammatory cytokines (e.g., tumor necrosis factor-α, interleukin-1β, interleukin-8, interleukin-6) [53]. Such cytokines suppressor effect of alphitolic acid (30) can help in reducing the severe inflammatory syndrome associated with COVID-19, which is the major cause of the high mortality rate reported for this disease [32].

Betulinic acid (BetA) (31) (Figure 6) is a pentacyclic lupine type triterpene, which has been reported to display broad-spectrum antiviral activity (e.g., HIV and HSV) [54]. This compound is recovered from the bark of birch trees and other pants e.g., leaves of *Sizigium claviflorum* in high quantity. Esterification of BetA has yielded some semisynthetic derivatives (e.g., 3-acyl derivatives and the ester analog 3-*O*-(3′,3′-dimethylsuccinyl) that were more potent inhibitors (EC_50_ 1.4 µM) against HIV-1. BetA antiviral mode of action was proposed to be by the interference with normal gag processing, which is crucial for the formation of mature infectious virus particles. The C-3 position of betulinic acid analogs was found to be a pharmacophore for anti-HIV maturation activity, and optimal potency, it was found that the analog should have a C-3 acyl side with the proper length, terminal carboxylic acid moiety, and dimethyl substitution at the C-3‘ position [55,56]. BetA derivatives substituted with ω-aminoalcanoic acid at the C-28 position showed higher anti-HIV activity in the nanomolar range via interfering with the virus-cell fusion process [57]. So, The C-28 position of BetA has been considered as a pharmacophore for inhibition of HIV entry. Additionally, the addition of the acyl side chain at C-3 and the amide side chain at C-28 to BetA resulted in derivatives that prohibited both HIV entry and maturation [58]. Betulinic acid-derived compounds, especially the 3,28-di-oacetylbetulin inhibited Semliki Forest virus (SFV) replication during plaque reduction assay [59]. One of the most important structural contributors to the anti-SFV activity is the free or acetylated OH group at C-3. The antiviral activity of betulinic acid and its derivatives also has been studied against influenza A, herpes simplex type 1 (HSV-1), and ECHO-6 enterovirus (EC_50_ 1.3 µM; EC_50_ 5.1 µM; EC_50_ 0.9 µM, respectively) [59,60]. It has been found that the addition of ureides or the C-28 amide group to betulinic acid increases its anti-HSV-1 activity [61,62]. Moreover, the 3-oxime derivatives of betulinic acid were more active than betulinic acid against the influenza A virus with EC_50_ of 2.8 µM [43], whereas betulinic aldehyde showed antiviral activity against the H9N2 subtype of the avian influenza virus [48]. Another betulinic acid derivative called 3β,28-di-onicotinoylbetulin is active against human papillomavirus (HPV) type 11 with an IC_50_ value of 12.4 µM type [49]. 

Dammaradienol, dammarenolic acid, hydroxydammarenone I, and hydroxyhopanone (32–35) (Figure 6) are four seco-dammarane type triterpenes that have been isolated from *Balanocarpus heimii* (Dipterocarpaceae) and *Dipterocarpus alatus* resin; the two compounds possess antiviral activity against HSV-1 with IC_50_ values equal to 2.5 µM, 3 µM, 2 µM, and 7 µM, respectively, and HSV-2 virus (3 µM, 2 µM, 5 µM, and 5 µM), respectively [33]. 

Glycyrrhizin (36) (Figure 6) is a common triterpenoid saponin glycoside found in *Glycyrrhiza glabra* roots. It was used as a sweetener and flavoring agent in folk medicine [63]. The antiviral activity of glycyrrhizin and its related compounds were studied against a large variety of viruses. Glycyrrhizin and its metabolites such as glycyrrhetic acid (GA), which is a pentacyclic triterpene belong to the β-amyrin type obtained by hydrolysis of glycyrrhizin, reported having the ability to inhibit the cytopathic effect of numerous DNA and RNA viruses such as vaccinia, HSV-1, Newcastle disease, and vesicular stomatitis virus (VSV) in vitro [64]. Glycyrrhizin has been also shown in vitro antiviral activity against the varicella-zoster virus [65] in human embryonic fibroblasts with IC_50_ equal 0.75 mM. Moreover, Glycyrrhizin has an inhibitory effect on human flaviviruses, such as Japanese encephalitis virus (JEV) dengue virus, tick-borne encephalitis virus (TBEV), West Nile virus (WNV), and many others (EC_50_ equal to 384 µM; EC_50_: 474 µM; EC_50_: 229 µM, respectively) [66]. It was also reported that glycyrrhetic acid is active against EBV via interference with EBV entry into the host cells [67]. In vitro studies showed that glycyrrhizin stopped the secretion of HBV surface antigen (HBsAg) from hepatocytes infected with HBV [68]. It also prohibited the viral cytopathic effect and the virus-specific antigen expression in HIV-infected MT-4 cells and inhibited virus replication in cultures of leukocytes from HIV-infected patients in IC_50_ equal to 0.3 mM) [69]. It worth mentioning that, glycyrrhizin can inhibit SARS CoV entry to Vero cells (EC_50_: 600 mg/L) [70] and H1N1 influenza virus uptake into human bronchial epithelial cells (EC_50_: 500 µM) [71]. The antiviral effect of glycyrrhizin against pseudorabies herpesvirus (PrV) was detected with IC_50_ values ranged between (0 and 125 µM) [72], while glycyrrhetic acid inhibited in vitro rotavirus replication, and affected the virus entry into the cells [73]. It has been shown recently that, glycyrrhizin lead to loss of proper mRNA production and to cause a defect in sister chromatid cohesion in IC_50_ equal to 2-4 mM, this process is necessary for both Kaposi‘s sarcoma-associated herpesvirus (KSHV) replication and cellular chromosome stability, which resulted in the inhibition of proliferation of B lymphocytes latently infected with KSHV [74]. Additionally, glycyrrhizin showed promising antiviral activity against the hepatitis C virus with IC_50_ of 5 µM in hepatic cells transfecting with HCV 3a core plasmid [75]. 

Pomolic acid (PA) (37) (Figure 6) is a pentacyclic ursane-type triterpenoid and is considered the1 9-hydroxy derivative of ursolic acid [76]. It has been isolated from a wide range of plants e.g., *Rosa woodsia* (Rosaceae) and *Hyptis capitata* (Labiatae), it was also identified as an anti-HIV agent (EC_50_ 1.4 µM). Studies showed that pomolic acid can also reduce carrageenan-induced paw edema in mice which indicates its strong anti-inflammatory activity. It can also inhibit the in vivo production of interleukin-1β by 39% [77]. 

Three quinone-methide triterpenes, pristimererin, tingenone, and iguesterin (38–40) (Figure 6) have been isolated from *Triterygium regelii*. The three compounds were evaluated for their SARS-CoV 3CLpro inhibitory activities and showed potent inhibitory activities with IC_50_ values of 5.5, 9.9, and 2.6 µM, respectively. Studies showed that the quinone-methide moiety in A-ring and increasing hydrophobicity in E-ring increase the virucidal potential [78]. Under the acidic conditions, the quinone-methide chromophore can rearrange to phenolic systems [79,80,81,82,83] and yield isomers which were found to have an inhibitory effect on HSV [84].

Saikosaponins (41) (Figure 6) is a group of oleanane derivatives usually present as glucosides, in many medicinal plants e.g., *Bupleurum* spp., *Heteromorpha* spp., and *Scrophularia scorodonia.* Saikosaponins (A, B_2_, C, and D) have been proved to be active against human immunodeficiency virus (HIV) [85], measles [86], influenza virus [77,78] HSV, and varicella-zoster virus [65] Saikosaponin A has been reported to possess anti-HCoV-229E activity with an IC_50_ value of 8.6 µM. Ursolic acid (UA) (42) (Figure 6) is a naturally occurring pentacyclic triterpene carboxylic acid, with various pharmaceutical properties present as a free acid or as saponins. UA was isolated from *Balanocarpus heimii*. UA was found to have antiviral activity in *in-vitro* against rotavirus infections and It inhibited rotavirus replication in IC_50_ of 10 µM [87]. It displayed partly anti-HCV activity through suppressing HCV NS5B RdRp activity as noncompetitive inhibitors. Therefore, ursolic acid is suggested to be used as potential HCV antivirals and can be applied to clinical trials either as monotherapy or in combination with other HCV antivirals [88].

## 5. In Silico Investigation 

**Figure 7:**
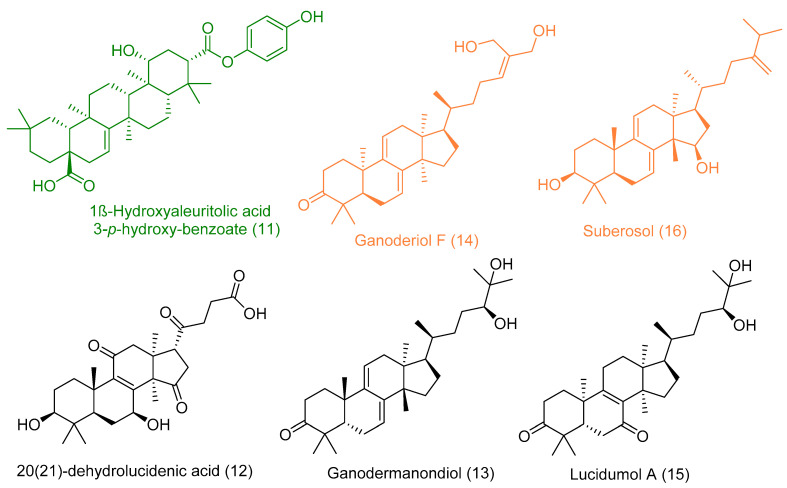
Top-scoring triterpenes retrieved from docking against both viral and human-based targets. Hits for 5 targets (Green color), hits for 2 targets (Orange color), and hits for only one target (Black color).

To suggest possible anti-SARS-CoV-2 candidates, the 162 compounds reviewed in this investigation were subjected to a docking study against both virus-based and human-based molecular targets (three and four targets, respectively) that their structures are currently available at protein data bank website (PDB, https://www.rcsb.org/). Six triterpenes were retrieved as top hits for one or more targets depending on the binding energies they scored (i.e., got binding energy lower than −6 kcal/mol) and their interactions with the reported key amino acid residues (Table 1 and Figure 7). 

Both SARS-CoV-2 main protease (M^pro^) and papain-like protease (PL^pro^) are key viral cysteine proteases essential for the viral replication inside the host cell, where they activate the replication complex that in turn initiate the viral RNA replication [15].

**Figure 8:**
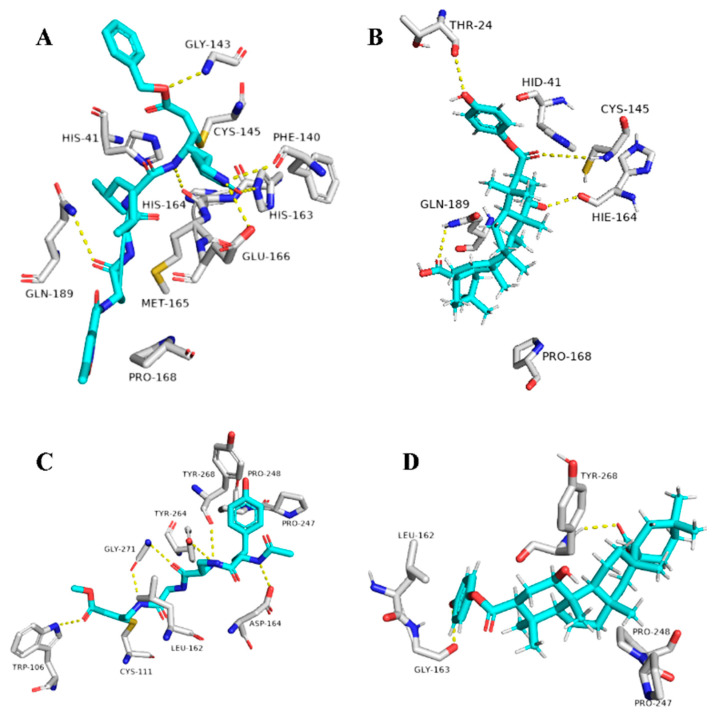
Binding modes of 1ß-Hydroxyaleuritolic acid 3-*p*-hydroxybenzoate (11) inside SARS-CoV-2 targets. (**B**,**D**) Interactions inside both M^pro^ and PL^pro^, respectively. (**A**,**C**) co-crystallized ligands of both M^pro^ and PL^pro^, respectively.

The triterpene 1β-hydroxyaleuritolic acid 3-*p*-hydroxybenzoate (11), which was previously isolated from the roots of *Maprounea Africana* [89], was the only compound that got interesting binding scores against the viral main protease (M^pro^) and papain-like protease(PL^pro^) (binding scores of −8.5 and -8.0 kcal/mol, respectively, Table X). This compound was able to form four strong hydrogen bonds (<2.5 Å) and with four different amino acid residues inside the M^pro^’s active site (Table 1 and Figure 8), three of them have been reported previously as essential ones for the enzyme’s inhibition [36]. Additionally, it was able to form two hydrophobic interactions with HID-41 and PRO-168 that have been also reported to be involved in the interactions with the co-crystallized inhibitors. Regarding PL^pro^, this compound achieved very good fitting inside the enzyme’s active site with 2 H-bond and 4 hydrophobic interactions with reported key amino acid residues (Table 1 and Figure 8) [90].

In addition to the viral target proteins, 1ß-Hydroxyaleuritolic acid 3-*p*-hydroxybenzoate was able to give interesting docking scores with many non-viral targets like adaptor protein 2 associated kinase (AAK1), furin, and cathepsin L (Binding energy = −9.1, −8.7, and −8.1 kcal/mol, respectively).

AAK1 together with cyclin G-associated kinase (GAK) are considered two key proteins involved in the viral endocytosis process, and hence inhibition of these kinases can prevent the viral entry inside the host cells [91]. On the other hand, furin and cathepsin L are two host-based proteases that are widely distributed in the human lungs. Both enzymes have been reported to be involved in the activation of the viral spike protein (S-protein), which is considered a key step in the viral-host cell attachment and subsequently, viral entry [92]. Thus, targeting these host-based proteins can provide potential anti-COVID-19 therapeutics.

**Figure 9:**
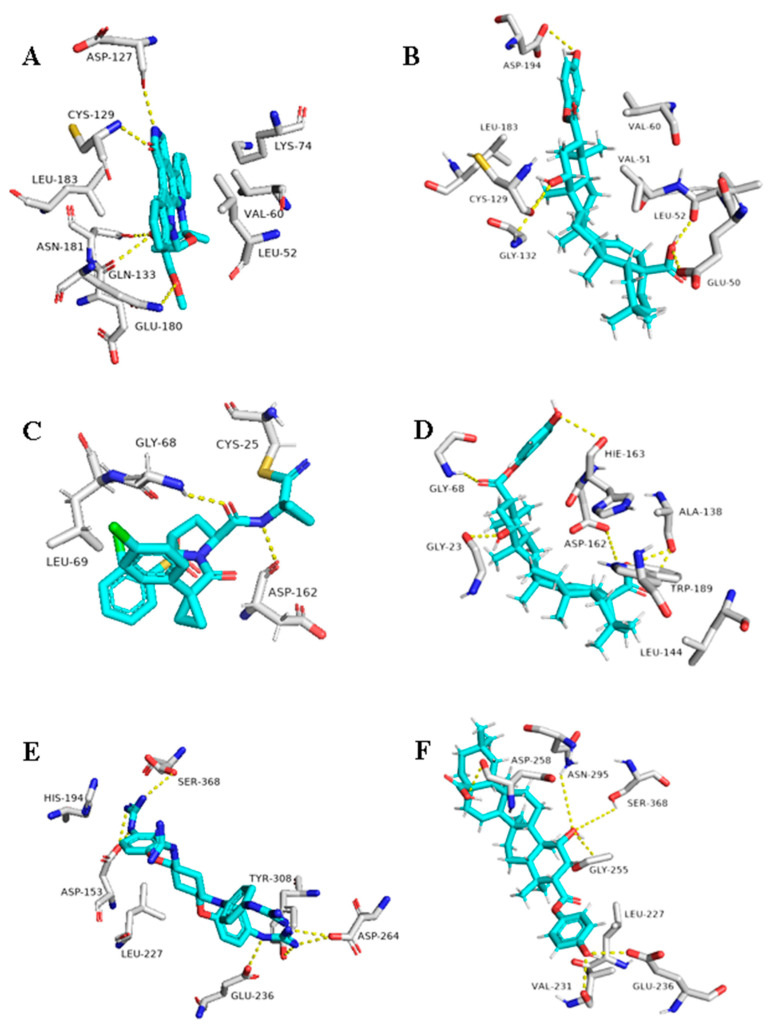
Binding modes of 1ß-Hydroxyaleuritolic acid 3-*p*-hydroxybenzoate (11) inside the human-based targets. (**B**,**D**,**F**) Interactions inside both AAK1, cathepsin L, and furin, respectively. (**A**,**C**,**E**) co-crystallized ligands of AAK1, cathepsin L, and furin, respectively.

As illustrated in Table 1 and (Figure 9), 1ß-Hydroxyaleuritolic acid 3-*p*-hydroxybenzoate (11) was able to fit itself inside the AAK1’s active site through 5 H-bonds with 5 different amino acid residues together with hydrophobic interactions with another three. Moreover, it accommodated itself inside the active sites of both furin and cathepsin L via the formation of 6 H-bonds. Interestingly, 1ß-Hydroxyaleuritolic acid 3-*p*-hydroxybenzoate (11) has been also found to be a potent inhibitor of HIV-1 reverse transcriptase and DNA polymerase with IC_50_ values of 3.7 and 7.4 µM, respectively [24] (Figure 9).

The next top scoring compound was ganoderiol F (14). It is an antitumor triterpene produced by *Ganoderma lucidum*, and its best binding scores were achieved against the viral ADP ribose phosphatase (ARP) and furin (−8.0 and −8.3 kcal/mol, respectively, Table 1). ARP was suggested to be involved in the viral protection against the ADP-ribosylation activated by the innate immune system of the host cells, and hence, this protein can be considered a relevant drug target [93,94].

**Figure 10:**
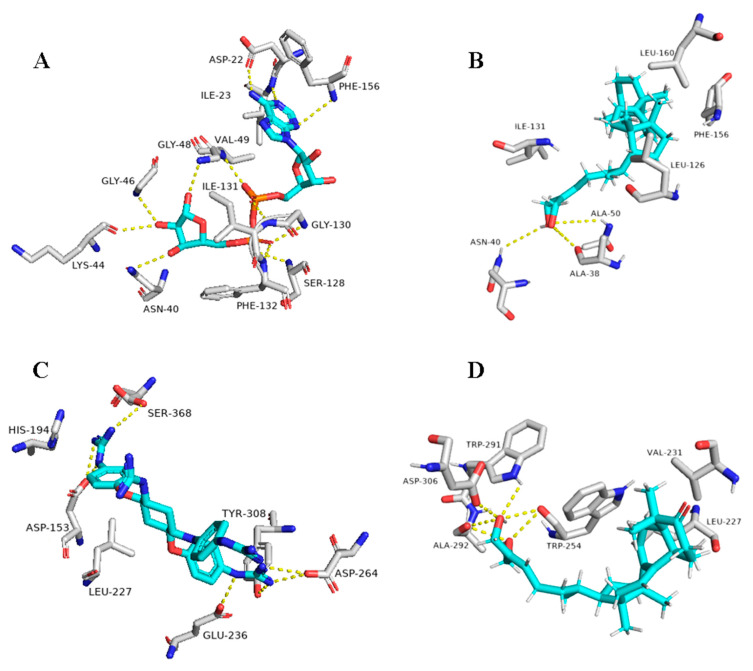
Binding modes of Ganoderiol F (14) inside the viral and human-based targets. (**B**,**D**) Interactions inside both ARP and furin, respectively. (**A**,**C**) co-crystallized ligands of ARP and furin, respectively.

Ganoderiol F (14) was able to form only three H-bonds inside ARP’s active site, however, it interacted with other four amino acid residues through hydrophobic interactions (Table 1 and Figure 10).

Unlike 1ß-Hydroxyaleuritolic acid 3-*p*-hydroxybenzoate (11), ganoderiol F (14) interacted with only four adjacent amino acid residues inside furin’s active site (Table 1 and Figure 10), however, its hydrophenanthren body was imbedded inside a hydrophobic pocket (LEU-227, VAL-231, and TRP-254).

**Figure 11:**
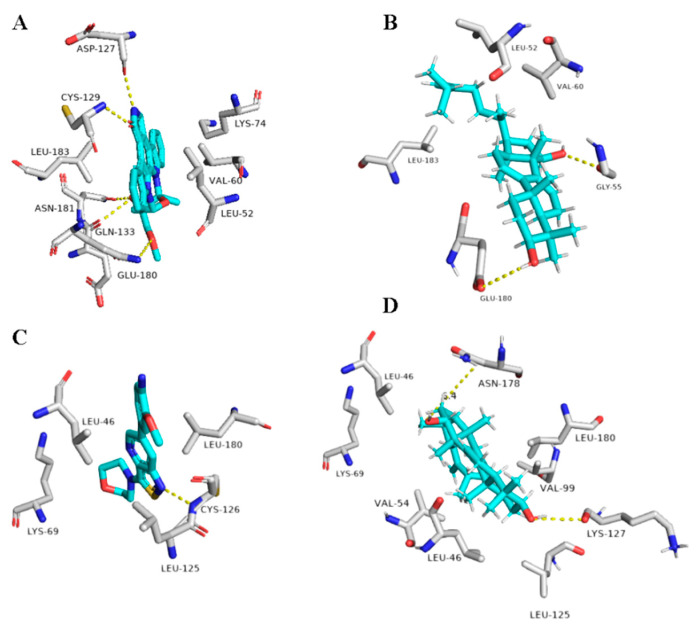
Binding modes of Suberosol (16) inside the human-based targets. (**B**,**D**) Interactions inside both AAK1 and GAK, respectively. (**A**,**C**) co-crystallized ligands of AAK1 and GAK, respectively.

Regarding the triterpene suberosol (16), which has been previously isolated from *Polyalthia suberosa* [50], and shown anti-HIV activity, was also able to get high docking scores against 2 targets, AAK1 and GAK (−9.0 and −8.2 kcal/mol, respectively). Both of these targets are human-based kinases involved in the viral endocytosis regulation [91]. Interestingly, this compound has been shown in vitro antiviral activity against HIV [50]. This compound was able to interact inside both AAK1 and GAKs active site via H-bond (2 interactions) and hydrophobic interactions (three and five interactions, respectively) (Table 1 and Figure 11). 

The *G. lucidum*-derived triterpene 20(21)-dehydrolucidenic acid (12) has been shown previously in vitro antiviral activity against both HIV and HCV [95]. In addition, it gave an interesting docking score with the human-based target GAK (Binding energy = −8.1 kcal/mol). Being a polyhydroxylated triterpene, 20(21)-dehydrolucidenic acid (12) was able to interact with six amino acid residues inside the enzyme’s active site through 9 H-bonds together with two hydrophobic interactions with VAL-54 and LEU-180 (Figure 11).

**Figure 12:**
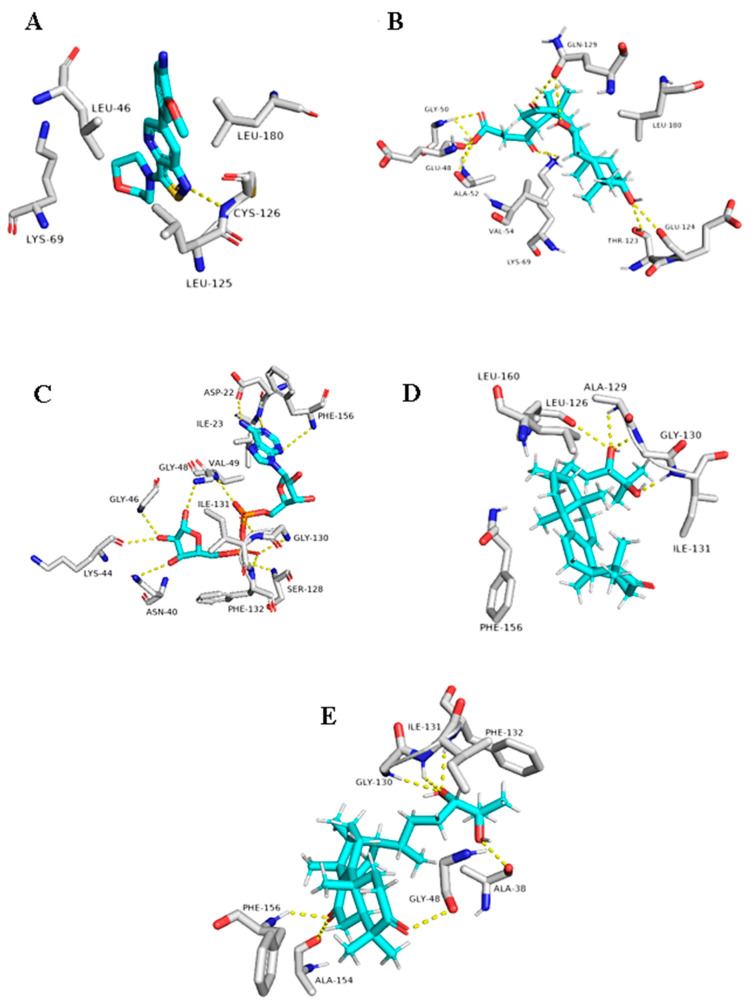
Binding modes of 20(21)-dehydrolucidenic acid (**B**) (12), Ganodermanondiol (**D**) (13), and Lucidumol A (**E**) (15) inside the viral and human-based targets (GAK and ARP, respectively). (**A**,**C**) co-crystallized ligands of GAK and ARP, respectively.

The last two triterpenes ganodermanondiol (13) and lucidumol A (15) have been also derived from *G. lucidum*, where the former compound has shown inhibitory activity against HIV (IC_50_ 90 µm) [96]. Both triterpenes sowed interesting binding energies against the viral target ARP (Binding energy = −8.3 and −9.6, respectively). Additionally, they interacted with most of the key amino acid residues [94], particularly, lucidumol A (15) (Table 1 and Figure 12).

Judging from the previous discussion, it can be concluded that the triterpene class of compounds, particularly, the polyhydroxylated ones, can be utilized in the development of multi-target inhibitors against COVID-19. Additionally, four top-scoring compounds out of six have been reported previously from *G. lucidum* indicating that this mushroom could be a valuable source and good starting point for exploring anti-SARS-CoV-2 therapeutics. 

## 6. Conclusions

Sterols and triterpenes comprise a huge class of compounds with different biological activities, where mainly they act as anti-cancer, anti-inflammatory, immunomodulatory, and anti-viral. In this review, we were focusing on the antiviral hits, in natural form or derivatized ones that showed potent activities as anti-HIV and showing up their mechanisms of activities that may link these compounds to be a potent hit for the treatment of COVID-19.

We can be concluded that some compounds can be a good hit for SARS-2, for example, 1β-Hydroxyaleuritolic acid 3-*p*-hydroxybenzoate (11) that acting as reverse transcriptase inhibitors, and at the same time it showed a protease inhibitor too; moreover, when the virtual docking of the previously collected compounds against different molecular targets, it was the only compound that got interesting binding scores against the viral main protease (M^pro^) and papain-like protease(PL^pro^) (binding scores of -8.5 and -8.0 kcal/mol, respectively). This compound was able to form four strong hydrogen bonds (< 2.5 Å) and with four different amino acid residues inside the M^pro^’s active site, three of them have been reported previously as essential ones for the enzyme’s inhibition which conformed to its powerful antiviral activity and can be considered a role model for inhibition of COVID-19. In the same manner, Lupeol (12) and Uvaol (14) showed powerful reverse transcriptase inhibitors. Salaspermic acid (13) showed inhibitory activity against HIV via reverse transcriptase and it has replication inhibition activity.

On the other hand, if we are talking about the replication inhibitors, we can conclude that β-Aescin (19) is the one of choice, as it was reported that it inhibits NF- *ƙ*B activation and cytokines production as well as, the inhibition of the replication of SARS-CoV-1 in vitro (IC_50_ 6 µM), suggest it may be able to inhibit SARS-CoV-2 too.

Some of the sulfated sterols e.g halistanol sulfate F (25) and halistanol sulfate G (26) exhibited antiviral activity against HIV-1, this may suggest the importance of the sulfate group in the inhibition of the virus. Alphitolic acid (30) showed an effect quite similar to that of dexamethasone in inhibition of the nuclear factor kappa-B (NF-κB), or suppressing the secretion of pro-inflammatory cytokines such as cytokines suppressor effect of alphitolic acid (30) can help in reducing the severe inflammatory syndrome associated with COVID-19.

The previous discussion showed that not only the triterpenes are active but also their semi-synthetic derivatives e.g., betulinic acid (31) as the antiviral activity of betulinic acid and its derivatives also have been studied against influenza A, herpes simplex type 1 (HSV-1), and ECHO-6 enterovirus. It has been found that the addition of ureides or the C-28 amide group to betulinic acid increases its anti-HSV-1 activity Moreover, the 3-oxime derivatives of betulonic acid were more active, and this increase the number of active hits and widen their activities.

One of the most common triterpenes is glycyrrhizin (36) and its metabolites, such as glycyrrhetic acid (GA), reported having the ability to inhibit the cytopathic effect of numerous DNA and RNA viruses such as vaccinia, HSV-1, Newcastle disease, and vesicular stomatitis virus (VSV) in vitro. In vitro studies also showed that glycyrrhizin stopped the secretion of HBV surface antigen (HBsAg) from hepatocytes infected with HBV and also prohibited the viral cytopathic effect and the virus-specific antigen expression in HIV-infected MT-4 cells and inhibited virus replication in cultures of leukocytes from HIV-infected patients and it worth mentioning that, glycyrrhizin can inhibit SARS CoV entry to Vero cells.

Concerning M^pro^ one of the most widespread targets for COVID-19, it was found the quinone-methide triterpenes, pristimererin, tingenone, and iguesterin (38–40) have shown potent inhibitory effect against SARS-CoV 3CLpro, this may contribute to the quinone group as through docking study ganoderiol F, that having similar quinone group in the same position showed the next scoring potency to the top 1β-Hydroxyaleuritolic acid 3-p-hydroxybenzoate (11).

Finally, we would like to highlight the activity of ursolic acid (UA) (42) that was found to have antiviral activity in-vitro against rotavirus infections and it inhibited rotavirus replication, this may suggest its ability to inhibit some of coronaviruses effects when attacking the GIT in similar mechanisms.

## Figures and Tables

**Table 1:** Binding free energies in kcal/mol of the top-scoring triterpenes against 7 viral and non-viral targets (3 viral-based and 4 human-based).

Compound	ARP (6W02)	M^pro^ (6LU7)	PL^pro^ (6WXR)	AAK1 (4WSQ)	GAK (4Y8D)	Cathepsin L(2YJC)	Furin (6EQX)
**1ß-Hydroxyaleuritolic acid 3-p-hydroxy-benzoate (11)**	>−4	−8.5	−8	−9.1	>-4	−8.1	−8.7
**20(21)-dehydrolucidenic acid (12)**	>−4	>−4	>−4	>−4	−8.1	>−4	>−4
**Ganodermanondiol (13)**	−8.3	>−4	>−4	>−4	>−4	>−4	>-4
**Ganoderiol F (14)**	−8	>−4	>−4	>−4	>−4	>−4	−8.3
**Lucidumol A (15)**	−9.6	>−4	>−4	>−4		>−4	>−4
**Suberosol (16)**	>−4	>−4	>−4	−9	−8.2	>−4	>−4
